# Calcification of Tooth Pulp

**Published:** 1869-02

**Authors:** 


### CALCIFICATION OF TOOTH PULP.

Recently Miss L——, aged twenty-one, of nervous, sanguine temperament and good general health, called for consultation in reference to her two superior central incisors. About four years before they had received a blow, which partially loosened them; they were quite sore and painful for a few weeks, and then recovered so far as to be used with a tolerable degree of comfort. The left tooth soon changed somewhat in color, and the presumption was that the pulps of both were devitalized. Two years and a half after the accident the teeth began to change position, the cutting edges being thrown forward against the upper lip, disfiguring the mouth very much.

In consequence of this, together with constant soreness, which had existed for several months, it was decided to remove them, which being done nothing peculiar was observable, further than had been shown before extraction, the left tooth showing some change of color, but the right none from that of a healthy tooth.

Through inadvertence, the crown of the latter, a day or two after extraction, was broken into three or four pieces, breaking off at the neck of the tooth; the pulp was found to be completely calcified, entirely filling the pulp chamber; it did not break; the fragments of the crown parted from it, leaving it standing perfect, tightly imbedded in the canal of the root so firmly that it can not be drawn out with the fingers. This is the only case of the kind we have ever seen, and is a very marked illustration of a process upon which very little attention has been bestowed, and about which not much is known.

We were recently presented by Dr. Cushing, of Chicago, with a section of tooth, in which the calcification of the pulp was complete, but it was perfectly united to and continuous with the dentine all round the walls of the pulp chamber, which was obliterated thereby.

This is clearly a calcification of the pulp, and not a deposition merely of calcific matter upon the walls of the chamber, for the structure of the pulp is clearly seen in the tissue. We shall have sections of each mounted for microscopic examination, when we shall perhaps have something further to say in reference to them. T.

